# Environmental and Occupational Respiratory Diseases – 1031. A study of outdoor aeroallergens in Riyadh, Saudi Arabia

**DOI:** 10.1186/1939-4551-6-S1-P30

**Published:** 2013-04-23

**Authors:** Syed Mohammed Hasnain, Salma Kabbara, Abdullah Saad Al-Modaihsh, Othman Mahjob

**Affiliations:** 1 Cell Biology, King Faisal Specialist Hospital and Research Centre, Riyadh, Saudi Arabia; 2 College of Agriculture, King Saud University and King Khalid University Hospital, Riyadh, Saudi Arabia

## Background

To investigate the outdoor aeroallergens at two different, a fully developed (FD) and a less developed (LD), sites in Riyadh, capital city of Saudi Arabia.

## Methods

Two Burkard Volumetric 7-day Recording Spore Traps were employed at both locations. Exposed slides were read at 12 bi-hourly traverses, with a total of 60 fields per 24 hours (0-24hours). The study was initiated in January 2012.

## Results

The data analyzed so far revealed the presence of different pollen and fungal species at both sites. The ratio between the pollen and fungal spores was 1:5. The airborne pollen grains showed quantitative and qualitative differences between the two sites. At FD site, the major pollen grains included *Phoenix dactylifera* (31%), grass pollen (14%), *Atriplex nummularia* (12%), *Amaranthus viridus* (10%), *Artemisia monosperma* (7%), *Plantago* sp. (6%) and *Populus* sp. (4%). The minor species (<1%) were *Casuarina* sp., *Chenopodium murale*, *Eschscholtzia* sp., *Eucalyptus* sp., *Lilium* sp., *Prosopis juliflora*, *Salix* sp. and *Salsola imbricata.* At the LD site, the major species included *Phoenix dactylifera* (36%), grass pollen (15%), *Salix* sp (8%), *Artemisia monosperma* (6%), *Atriplex nummularia* (6%), *Plantago* sp. (3%), *Populus* sp. (3%), *Amaranthus viridus* (2%) and *Ambrosia* sp. (2%). The minor species included *Daucus carota*, *Eucalyptus* sp. and *Salsola imbricata*. The fungal spores recorded at both sites displayed only quantitative variations. The major components of these spores at FD site included *Arthrinium* (14%), *Ulocladium* (14%), *Cladosporium* (13%), *Alternaria* (10%), ascospore septate (10%), smut spores (9%), *Basidiospore* (7%), ascospore non septate (6%), *Bipolaris* (3%), *Myxomycete* (3%), *Periconia* (3%), *Aspergillus/Penicillium* (2%), *Stemphyllium* (2%) and *Curvularia* (1%). The minor components (<1%) were *Drechslera*, *Melanospora*, *Peranospora*, *Pithomyces* and *Torula*. The major fungal spores at LD site were *Ulocladium* (13%), *Cladosporium* (12%), *Aspergillus/Penicillium* (11%), smut spores (11%), *Alternaria* (9%), ascospore septate (9%), *Basidiospore* (9%), *Arthrinium* (8%), *Stemphyllium* (5%), ascospore non septate (4%), *Periconia* (3%) and *Bipolaris* (2%), while the minor spores included *Curvularia*, *Drechslera*, *Melanospora*, *Myxomycete*, *Peranospora*, *Pithomyces* and *Torula.*

## Conclusions

The results indicate that developed and less developed sites have no impact on the qualitative and quantitative diversity of fungal spores but such diversities were recorded in airborne pollen species.

